# A qPCR to quantify *Wolbachia* from few *Onchocerca volvulus* microfilariae as a surrogate for adult worm histology in clinical trials of antiwolbachial drugs

**DOI:** 10.1007/s00436-021-07411-5

**Published:** 2022-01-10

**Authors:** Stefan Schlabe, Patricia Korir, Christine Lämmer, Frederic Landmann, Bettina Dubben, Marianne Koschel, Anna Albers, Linda Batsa Debrah, Alexander Yaw Debrah, Marc P. Hübner, Kenneth Pfarr, Ute Klarmann-Schulz, Achim Hoerauf

**Affiliations:** 1 Department of Internal Medicine I, University Hospital Bonn, Bonn, Germany; 2 German Center for Infection Research (DZIF), Partner Site Bonn-Cologne, Bonn, Germany; 3 Institute of Medical Microbiology, Immunology and Parasitology, University Hospital Bonn, Bonn, Germany; 4 Centre de Recherche de Biologie Cellulaire de Montpellier (CRBM), Université de Montpellier, CNRS, 34293 Montpellier, France; 5 Kumasi Centre for Collaborative Research in Tropical Medicine (KCCR), Kumasi, Ghana; 6 Department of Clinical Microbiology, Kwame Nkrumah University of Science and Technology, Kumasi, Ghana; 7 Faculty of Allied Health Sciences of Kwame, Nkrumah University of Science and Technology, Kumasi, Ghana

**Keywords:** qPCR, *Wolbachia*, *Onchocerca volvulus*, *Litomosoides sigmodontis*

## Abstract

The filarial nematode *Onchocerca volvulus* causes onchocerciasis (river blindness), a neglected tropical disease affecting 21 million people, mostly in Sub-Saharan Africa. Targeting the endosymbiont *Wolbachia* with antibiotics leads to permanent sterilization and killing of adult worms. The gold standard to assess *Wolbachia* depletion is the histological examination of adult worms in nodules beginning at 6 months post-treatment. However, nodules can only be used once, limiting the time points to monitor *Wolbachia* depletion. A diagnostic to longitudinally monitor *Wolbachia* depletion from microfilariae (MF) at more frequent intervals < 6 months post-treatment would accelerate clinical trials of antiwolbachials. We developed a TaqMan qPCR amplifying the single-copy gene *w*Ov*ftsZ* to quantify *Wolbachia* from as few as one MF that had migrated from skin biopsies and compared quantification using circular and linearized plasmids or synthetic dsDNA (gBlock®). qPCR for MF from the rodent nematode *Litomosoides sigmodontis* was used to support the reproducibility and validate the principle. The qPCR using as few as 2 MF from *O. volvulus* and *L. sigmodontis* reproducibly quantified *Wolbachia*. Use of a linearized plasmid standard or synthesized dsDNA resulted in numbers of *Wolbachia*/MF congruent with biologically plausible estimates in *O. volvulus* and *L. sigmodontis* MF. The qPCR assay yielded a median of 48.8 (range 1.5–280.5) *Wolbachia*/*O. volvulus* MF. The qPCR is a sensitive tool for quantifying *Wolbachia* in a few MF from skin biopsies and allows for establishing the qPCR as a surrogate parameter for monitoring *Wolbachia* depletion in adult worms of new antiwolbachial candidates.

**Supplementary Information:**

The online version contains supplementary material available at 10.1007/s00436-021-07411-5.

## Introduction

*Onchocerca volvulus* nematodes cause onchocerciasis, a neglected tropical disease (NTD) known as river blindness, affecting 21 million people in Sub-Saharan Africa and remaining foci in Latin America. While Mass Drug Administration (MDA) was established to reduce transmission by targeting microfilariae (MF), elimination may not be possible for all endemic areas using MDA alone due to persisting, long-lived adult worms and reported suboptimal responses (Turner et al. 2013; WHO 2020). To achieve the WHO elimination goals by 2030, medicines that permanently sterilize or kill adult worms and sensitive diagnostics are needed. To address the first, antiwolbachial therapies, e.g., doxycycline, targeting the essential Gram-negative *Wolbachia* endosymbionts, provide an effective tool, preventing microfilariae (MF)-death-induced inflammation and adverse events and killing adult filariae within 2 years (Debrah et al. 2015; Hoerauf et al. 2001; Hoerauf et al. 2008; Klarmann-Schulz et al. 2017). To overcome the long treatment time with doxycycline (4–6 weeks), new compounds have been sought and several are entering phase 1 and 2 trials (Hübner et al. 2019, 2020; Krücken et al. 2021; Taylor et al. 2019; von Geldern et al. 2019; Hong et al. 2019). Even with nucleic acid amplification tests, one major obstacle to the development of new antiwolbachial drugs is that the treatment protocols lack an adequate early endpoint monitoring *Wolbachia* reduction necessary for go/no-go decisions. A significant effect on the adult worms can first be evaluated by histology after 18–24 months (Debrah et al. 2015; Hoerauf et al. 2008), with *Wolbachia* depletion preceding the macrofilaricidal effect (Hoerauf et al. 2001, 2008), a parameter analyzed together with the adult worm viability and therefore usually performed at the primary study endpoint (Klarmann-Schulz et al. 2017). Moreover, diagnostics targeting adult worms rely on surgical extirpation of the nodules, limiting the number of time points that can be examined. A diagnostic tool using MF emerged from skin snips would enable frequent and longitudinal monitoring and evaluation of the dynamics of the *Wolbachia* depletion.

We developed a qPCR protocol diagnostic tool for absolute quantification of *Wolbachia* in few MF by using commercially manufactured DNA fragments (gBlocks®) as qPCR standards. The qPCR allows longitudinal monitoring of *Wolbachia* at timepoints from treatment start to nodule extirpation and can be used to evaluate early treatment effect in clinical trials of antiwolbachial treatments.

## Material and methods

### Samples

*O. volvulus* MF were collected from MF that had migrated out of skin snips (Hoerauf et al. 2008). MF were counted in 24-well plates in which the initial DNA extraction step was also performed. DNA was extracted using a modification of the Qiagen DNA QiaAmp Micro Kit protocol using a minimum elution volume of 25 µL that was incubated on the column twice for 5 min. The samples were derived from three different antiwolbachial clinical trials (DOLF, ISRCTN50035143 (Batsa Debrah et al. 2020); MoRiOn, ISRCTN43697583; and AWOL-Mino, ISRCTN06010453 (Klarmann-Schulz et al. 2017)). An MF positive control to calculate the intraassay and interassay variability and stability after long-term storage was made by pooling 2517 MF from 11 archived samples from untreated participants (DOLF, ISRCTN50035143 (Batsa Debrah et al. 2020)).

### qPCR assay

The qPCR amplifies the single-copy gene *ftsZ* from *Wolbachia* (*w*Ov*ftsZ*; GenBank:AJ276501) and actin from *O. volvulus* (OvActin; GenBank:M84916.1). For standardization studies and technical performance analyses, we used a duplex qPCR for MF from the rodent filarial nematode *L. sigmodontis* (Hübner et al. 2019). Both qPCRs used TaqMan probes. Standard curves were created using a 1:10 dilution series of the mixed standards from 10^7^ copies/µL to 10^0^ copies/µL. All primers were synthesized by Microsynth AG, Balgach, Switzerland; TaqMan probes were synthesized by Biomers GmbH, Ulm, Germany (Table 1). Absolute quantification was done as a duplex qPCR with a reaction volume of 20 µL in a RotorGene 6000 (Qiagen, Hilden, Germany): 1xQuantiNova master mix (Qiagen), 400 nM for each forward and reverse primer, and 25 nM and 50 nM hybridization probe for *w*Ov*ftsZ* and OvActin, respectively, with activation at 95 ℃ for 2 min, followed by 45 cycles of 95 ℃ for 5 s and amplification at 58 ℃ for 30 s with fluorescence acquired on FAM (green) and JOE (yellow) channels at 58 ℃. All samples were measured in triplicate. Analysis of qPCR data was done using the RotorGene 6000 Series Software ver. 1.7 with normalization on control reactions (no-template controls, NTC). Quantification was determined by setting the fluorescence threshold at 0.01. Outlier removal was set to 15% (only signals 15% higher than background were considered true).

**Table 1:** Primers, probes, and gBlock**®** sequences

Primers	5’-3’	Tm℃
*w*Ov*ftsZ*FW	AGGAATGGGTGGTGGTACTG	60.5
*w*Ov*ftsZ*RV	CTTTAACCGCAGCTCTTGCT	58.4
OvActin FW	GTGCTACGTTGCTTTGGACT	58.4
OvActin RV	GTAATCACTTGGCCATCAGG	58.4
*w*Ls*ftsZ*FW	CGATGAGATTATGGAACATATAA	55.6
*w*Ls*ftsZ*	TTGCAATTACTGGTGCTGC	55
LsActin FW	ATCCAAGCTGTCCTGTCTCT	58.4
LsActin RV	TGAGAATTGATTTGAGCTAATG	54.8
Probes	5’-3’	
*w*Ov*ftsZ*TQP	6-FAM CCTTGCCGCTTTCGCAATCAC DDQ1	61
OvActinTQP	HEX AACAGGAAATGGCAACTGCTGC BHQ-1	58
*w*Ls*ftsZ* TQP	6-FAMCAGGGATGGGTGGTGGTACTGGAA TAMRA	63
LsActinTQP	HEX ACTACCGGTATTGTGCTCGATT TAMRA	52
gBlock®		
*w*Ov*ftsZ*	CATATAAAAGATAGTCATATGCTCTTTATTACAGC **AGGAATGGGTGGTGGTACTGGTACAGGTGCAGCACCAGTGATTGCGAAAGCGGCAAGGGAAGCAAG** **AGCTGCGGTTAAAG**ATAAAATGTTAAAAGAGAAAAAGATATTGACTGTT^#^	
OvActin	TTGTTCGTGACATCAAAGAAAAGCT **GTGCTACGTTGCTTTGGACTTCGAACAGGAAATGGCAACTGCTGCATCGTCATCGTCTCTCGAAA** **AATCTTATGAATTGCCTGATGGCCAAGTGATTAC**CGTAGGCAACGAACGATTTCGATGCC^#^	

^#^Target sequence is in bold. The underlined sequences are flanking regions from the genomic sequence to reach the gBlock**®** minimum length of 150 bp

Plasmid standards containing the *w*Ov*ftsZ* sequence were tested in two different conformations: as supercoiled plasmids and after linearization with the single-cutting restriction NotI-HF enzyme according to the manufacturer’s protocol (NEB, Ipswich, MA). For absolute quantification, we designed artificial dsDNA fragments (gBlock®, Integrated DNA Technologies, Coralville, Iowa, USA) (Table 1), dissolved in AE buffer to yield a final concentration of 1.2*10^10^ copies/µL. For quantification assays, a standard curve of 1.2*10^5^ to 1.2*10^0^ copies/µL was used.

### Immunofluorescence picture of *L. sigmodontis* MF

To determine the number of *Wolbachia* in a single MF, we fixed and stained *L. sigmodontis* MF (Landmann et al. 2012). The *Wolbachia* in the MF were labeled with anti-*Wolbachia* Surface Protein monoclonal antibody (1:500, NR-31029, obtained through BEI Resources, NIAID, NIH) (Fattouh et al. 2019) and detected with a rabbit anti-mouse antibody tagged with Alexa fluor 488 nm (Invitrogen, 1:500). MF nuclei were stained with DAPI (Serbus et al. 2012). MF were observed with a 63X objective on a Leica SP5 confocal microscope.

## Results and discussion

### Linear plasmid standards are required for absolute quantification of *Wolbachia* in *L. sigmodontis* MF

**Fig. 1:**
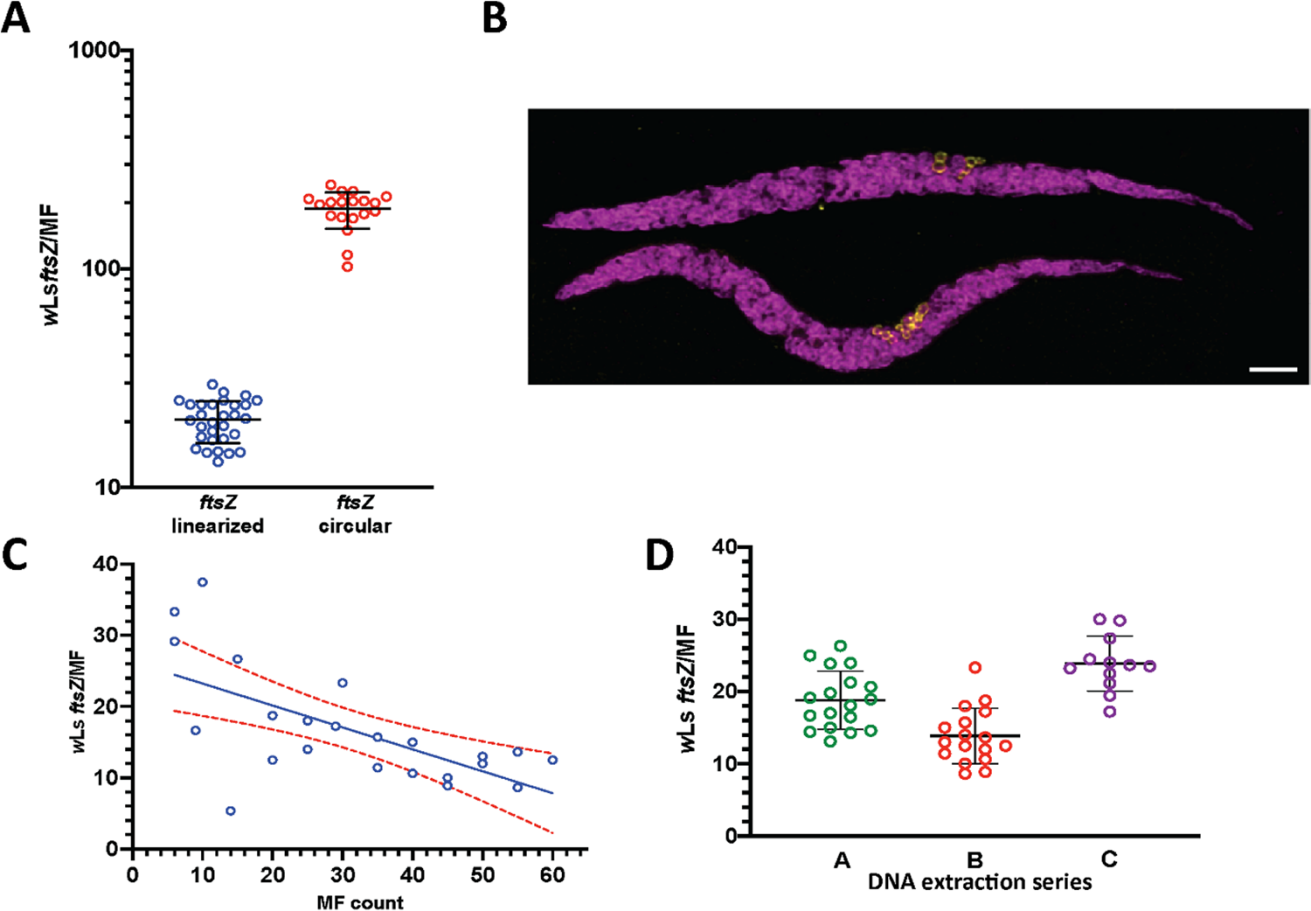
Supercoiled plasmid standards overestimate *w*Ls*ftsZ* copy numbers. (**A**) *L. sigmodontis* MF were counted (range 17 to 600) and DNA extracted for *w*Ls*ftsZ* qPCR. Quantification was done using circular (*N* = 19) or NotI linearized (*N* = 30) plasmids. Linearized plasmids resulted in reduced copy numbers (20.6 vs. 200 *w*Ls*ftsZ*/MF). Lines indicate median and interquartile range. (**B**) Full projection of confocal images showing 2 *Litomosoides sigmodontis* microfilariae stained with DAPI (magenta) and an anti-WSP (yellow) monoclonal antibody. Clusters of *Wolbachia* are located in the proximal part of the posterior half of MF. A single *Wolbachia* bacterium is 0.8–1 µm in length; thus, there are not hundreds of endobacteria in a single MF. MF were observed with a 63X objective on a Leica SP5. The scale bar = 10 µM. (**C**) Variation in *w*Ls*ftsZ* copy number per *L. sigmodontis* MF is greater in samples with few MF. DNA was extracted from 5 to 60 *L. sigmodontis* MF and used for *w*Ls*ftsZ* qPCR. Linear regression (blue line) and 95% confidence bars (red dotted lines) are shown. (**D**) The qPCR is reproducible and stable. Interassay reproducibility was tested on DNA extracted from 18 preparations, each with (**A**) 50 MF and (**B**) 20 MF, and (**C**) 12 preparations with 50 MF from *L. sigmodontis* to calculate *w*Ls*ftsZ* copies/MF. Lines indicate median and interquartile range

We established absolute quantification of *Wolbachia* per MF using *L. sigmodontis* MF. Parallel qPCR runs of NotI linearized and circular plasmid standards resulted in a tenfold difference in *Wolbachia* number, with 20.6 *w*Ls*ftsZ*/MF calculated using a linearized plasmid vs. 200 *w*Ls*ftsZ*/MF when using the circular (Fig. 1A). The difference in *Wolbachia* numbers was most likely due to less efficient amplification using supercoiled plasmid standards (Hou et al. 2010; Lin et al. 2011). To support that the linearized plasmid standard curve resulted in the biologically relevant number of *Wolbachia* in an MF, we compared the numbers provided by the qPCR with immunofluorescence images of *L. sigmodontis* MF (Fig. 1B). Using immunofluorescence, the MF clearly contained fewer than 200 *Wolbachia*. Thus, we concluded that the quantification using the linearized plasmid was more accurate.

To assess the performance of the qPCR on samples with different MF counts, including only a few MF, the DNA extraction and qPCR were systematically analyzed with duplicate samples of 5 to 60 MF. *w*Ls*ftsZ* copy number varied in samples with low MF counts, whereas samples with > 20 MF had calculated values within the 95% confidence bars (Fig. 1C). Nevertheless, all calculated *Wolbachia* numbers were within a biologically realistic range (5–39). In 3 independent extractions of MF from the same animal, the qPCR had good interassay reliability of *Wolbachia*/MF counts with a median between 14 and 25 (range 8–28) (Fig. 1D). The interassay reliability was confirmed using a second MF population (Supplementary Table 1).

### *O. volvulus Wolbachia* qPCR in MF is a surrogate for adult worm histology

Operable onchocercomata are a limited resource in drug trials, require invasive operations, and can be performed infrequently to provide *Wolbachia* histology, usually at the start and end of the study. The main objective in the establishment and validation of the qPCR was, therefore, the generation of a reliable method to assess *Wolbachia* reduction early in clinical trials that can be easily and frequently collected without the need for surgically obtained adult worms. Skin snips, which are less invasive and logistically easier to perform, can be taken at many timepoints throughout a study to monitor *Wolbachia* densities in MF (e.g., pre-treatment, during treatment, and every 2 months post-treatment follow-up) and generate robust data on the dynamics of *Wolbachia* depletion and possible rebounds when applying suboptimal treatments. In prior trials, histology of nodules was done at 6 months, 20 months, and 27 months after therapy, and it was shown that the *Wolbachia* depletion in adult worms seen at 20 months was already established at month 6 (ISRCTN71141922 and ISRCTN68861628) (Batsa-Debrah et al. 2017; Hoerauf et al. 2008; Specht et al. 2009). It must, however, be stated that while *Wolbachia* reduction by 2 logs, in past trials, has always preceded the treatment success confirmed by histology of the adult worms, parasitological success cannot be predicted definitely by *Wolbachia* depletion at months 6 or earlier, as these numbers may later rebound if treatment has been suboptimal. Based on human and animal trials, a sustained *Wolbachia* depletion by a threshold of at least 2 logs is required for female adult worm sterilization and macrofilaricidal effects (Albers 2011; Hong et al. 2019; Hübner et al. 2019; Specht et al. 2018; Turner et al. 2006).

**Fig. 2:**
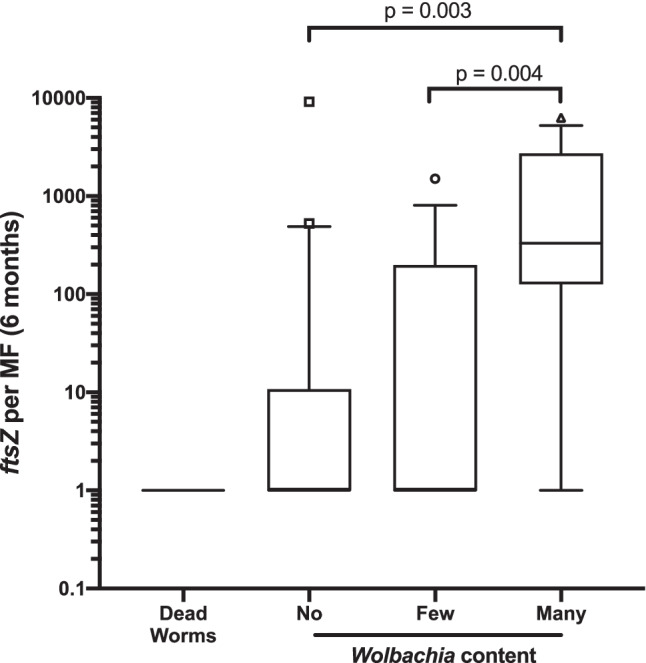
*w*Ov*ftsZ Wolbachia* qPCR of MF isolated from skin snips correlates with histological assessment of *Wolbachia* content in adult worms. The *Wolbachia* levels in adult worms at 6 months after treatment were divided into three classifications (no (*N* = 20), few (*N* = 17), many (*N* = 12) *Wolbachia* or 1 dead worm) by histology. *w*Ov*ftsZ* was measured by qPCR in the MF that had emerged from skin snips taken from the corresponding patients in the 6 months follow-up. There was a significant difference between the four groups regarding the *Wolbachia w*Ov*FtsZ*/MF (*p* = 0.003, Kruskal–Wallis test) with the main differences between many and no *Wolbachia* as well as many and few *Wolbachia* (*p* = 0.003, *p* = 0.004, respectively, Mann–Whitney *U*-test, SPSS version 24

To correlate *Wolbachia/*MF with the antiwolbachial effect in the adult worms, we measured the *w*Ov*ftsZ* levels in MF and compared the result to the *Wolbachia* levels in the adults assessed by histology in a recent trial on different regimens with doxycycline and rifampicin (ISRCTN06010453 (Klarmann-Schulz et al. 2017)). The *Wolbachia* depletion from adult worms had been determined by histology at 6 months after treatment in that trial. The *Wolbachia* levels of the MF from adult worms were divided into four groups: dead worms and many, few or no *Wolbachia*. The qPCR was done on DNA extracted from MF collected 6 months after treatment. The *Wolbachia/*MF determined by qPCR significantly correlated with groups of many, few, and no *Wolbachia* (Fig. 2). There was a significant difference between the four groups regarding the *Wolbachia w*Ov*FtsZ*/MF (*p* = 0.003, Kruskal–Wallis test) with the main differences between many (median 332 *w*Ov*ftsZ* copies/MF; IQR 128, 2665) and no *Wolbachia* (median 1 *w*Ov*ftsZ* copies/MF; IQR 1, 20; *p* = 003, Mann–Whitney *U*-test) as well as between many (median 332 *w*Ov*ftsZ* copies/MF; IQR 128, 2665) and few *Wolbachia* (median 1 *w*Ov*ftsZ* copies/MF; IQR 1, 193; *p* = 0.004, Mann–Whitney *U*-test). Thus, 6 months after treatment, the MF qPCR confirmed the *Wolbachia* depletion from adult worms determined by histology, validating the quantification of *Wolbachia* depletion from MF from skin snips as a suitable surrogate parameter for adult worm histology so that nodulectomies at this time point can be spared.

### The *O. volvulus Wolbachia* qPCR is sensitive for absolute quantification and longitudinal monitoring

**Fig. 3:**
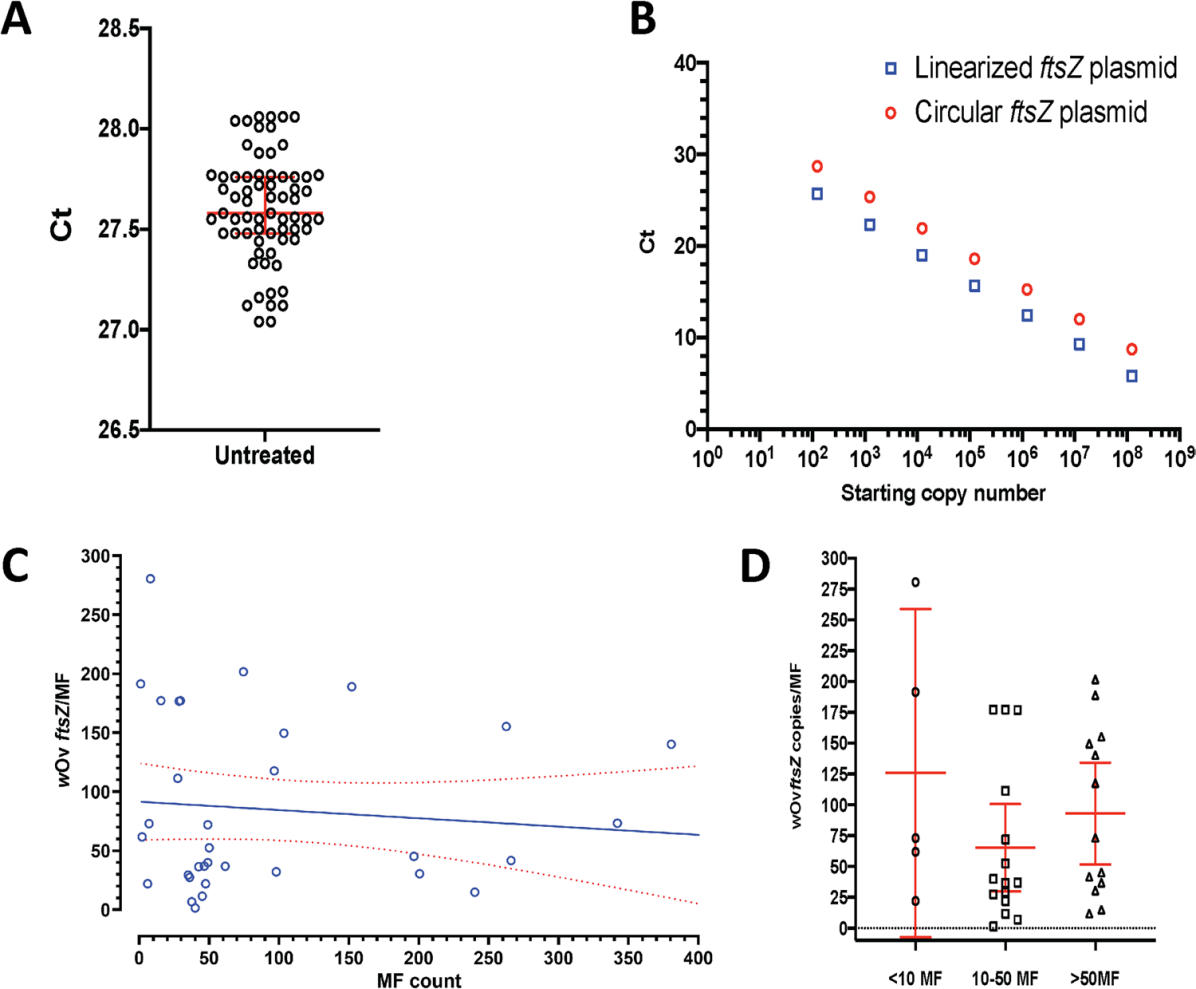
qPCR on *O. volvulus Wolbachia* show reliable quantification with linearized DNA standards. (**A**) Technical reproducibility of qPCR was performed using a positive control sample containing DNA from pooled MF samples from untreated patients, 67 qPCR runs were performed over a period of 1 year. Threshold cycle (Ct) varied ≤ 1 cycle. Lines indicate median and interquartile range. (**B**) Circular and NotI linearized plasmid standards differed by approximately 3 Ct, leading to an eightfold overestimation of *w*Ov*ftsZ*/µL using circular plasmid standards. (**C**) Variation of *w*Ov*ftsZ* copies/MF is dependent on the MF count of the preparation. DNA was extracted from 33 MF positive samples and used for qPCR with gBlock® standards. Samples with few MF resulted in a wider range and higher calculated *w*Ov*ftsZ*/MF. Linear regression (blue line) and 95% confidence bars (red dotted lines) are shown. One sample with 693 MF was omitted from the graph. (**D**) Determination of a cutoff for quantification was done by comparison of the 95% confidence intervals (CI) of the means in three different MF-count groups. DNA was extracted from 33 MF positive samples and used for qPCR with gBlock® standards. The samples were separated into three groups: < 10 MF, 11–50 MF, and > 50 MF, and the means and 95% CI were calculated (red lines) with GraphPad Prism version 9

Similar to *L. sigmodontis*, the copy number calculations done with the circular plasmid standard resulted in implausibly high *Wolbachia* counts in *O. volvulus*, i.e., several thousand *Wolbachia* per MF (Fig. 2). Such numbers were never seen by immunohistology and electron microscopy (Büttner et al. 2003; Martinez-Palomo and Martinez-Baez 1977). Nevertheless, the qPCR assay had a low variation of just 1 Ct in 67 runs performed over one year using aliquots of the same positive control sample (Fig. 3A). Use of NotI linearized plasmid as the template for a standard curve resulted in a shift in Ct of 3 cycles leading to eightfold lower *w*Ov*ftsZ* copy number compared to circularized plasmids (Fig. 3B, Supplementary Fig. 1). Attempts were made to do immunofluorescence in *O. volvulus* MF, but the fixation in ethanol in the field did not allow for good freeze-cracking and subsequent staining with the anti-WSP antibody.

To remove errors introduced by the preparation of plasmid DNA as standards, e.g., errors introduced by method of DNA quantification (Supplementary Fig. 2), the target sequences were synthesized as gBlock® (Conte et al. 2018). Because the gBlock® concentration was determined by HPLC, the numbers calculated from this standard are more accurate than other methods of DNA quantification. The reliable limit of detection was 10 *w*Ov*ftsZ*/µL (Supplementary Table 2). The qPCR could detect as few as 1 *w*Ov*ftsZ*/µL, but the variation of the triplicates was too great or not all replicates gave a signal or were close to background levels with Ct near 35 (Supplementary Table 2). The assay using gBlocks® was performed on 34 samples with a range of MF counts (1 to 693) per DNA extraction. This resulted in a median 48.8 *w*Ov*ftsZ*/MF (range 1.5–280.5, IQR 117.6) (Fig. 3C)).We concluded that the copy number calculated from the linearized plasmid/gBlock® was correct and was < 200 *Wolbachia*/MF. In the same sample set, the median actin copy number calculation was 1241.4 copies/MF (range 169.2–4111.8, IQR 1087.9). We do not know the number of cells per MF; an estimation can be 500 diploid cells. The median actin copy number calculation of our qPCR is biologically plausible, as the actin primers and probe will amplify both actin 1 and 2. The variation of the *Wolbachia* densities in this set of MF samples is obvious. We assume that the major contribution to this variation is caused by biological variation, different *Wolbachia* numbers in MF populations, which vary in adult worm age.

The *w*Ov*ftsZ*/MF variation was larger when using DNA extracted from low numbers of MF, in which one of three triplicates would produce a *Wolbachia* signal from 1 MF, whereas all samples with ≥ 2 MF provided a positive signal (Fig. 3C). To select a cutoff for quantification, we binned the MF counts as < 10, 10–50, and > 50 and compared the spread in 95% CI of the mean (Fig. 3D). The 10–50 MF samples had a CI spread that was equivalent to that of > 50 MF (71 copies/MF and 82 copies/MF, respectively), while the CI spread from < 10 MF was 266.3, almost the same as the range. Thus, ≥ 10 MF are needed to quantify the efficacy of antiwolbachial drugs in clinical trials.

Despite the large range of the *Wolbachia* densities, we expect to be able to evaluate a 2-log reduction under treatment for two reasons. First, the CI spread was smaller using a minimum of 10 MF. Second, it was regularly observed in preclinical trials that the range of *Wolbachia* densities in adult worms becomes smaller after treatment. This was observed with tetracyclines, rifamycins, and chinolones (Specht et al. 2018) and the new candidate ABBV-4083 (Hübner et al. 2019), but not with a suboptimal treatment regimen. We are confident to reliably confirm the effectivity of antiwolbachial treatment. In some cases, especially after successful antiwolbachial treatment, the MF from two or more skin snips of a participant could be pooled to achieve the minimum of 10 MF for DNA extraction. However, as a general rule, clinical studies would recruit participants with microfilaridermia high enough to achieve the minimum just using one skin snip.

## Conclusion

The qPCR on skin snip MF provides reliable quantification of *Wolbachia* in MF and can be used as a surrogate assay for immunohistology of *Wolbachia* in adult worms in nodules extirpated post-treatment that is less invasive, less expensive, and, most importantly, can use easily accessible skin snips. To provide a long-term storable and well-characterized standard for longitudinal monitoring, we selected gBlock® synthetic DNA. The use of the gBlock® standard curve fulfilled the requirements for sensitivity, reproducibility, and stability of the qPCR assay. The qPCR can evaluate *Wolbachia* densities at more frequent intervals, e.g., during treatment and 2-, 4-, and 6-months post-treatment to monitor *Wolbachia* depletion longitudinally and gain a more stable assessment of the *Wolbachia* dynamics to accelerate clinical trials of antiwolbachial therapies.

## Supplementary Information

Below is the link to the electronic supplementary material.

Supplementary file1 (DOCX 1600 KB)

## Data Availability

Not applicable.
