# Supplementary material for: A qPCR to quantify Wolbachia from few Onchocerca volvulus microfilariae as a surrogate for adult worm histology in clinical trials of antiwolbachial drugs
Source: Parasitol Res. 2022 Jan 10;121(4):1199–206. doi: 10.1007/s00436-021-07411-5 (PMC8986682; doi:10.1007/s00436-021-07411-5)
Supplement: Supplementary file 1 — Supplementary file1 (DOCX 1600 KB) [file 436_2021_7411_MOESM1_ESM.docx]

A qPCR to quantify Wolbachia from few *Onchocerca volvulus* microfilariae as a surrogate for adult worm histology in clinical trials of antiwolbachial drugs – supplemental material

Supplemental figures


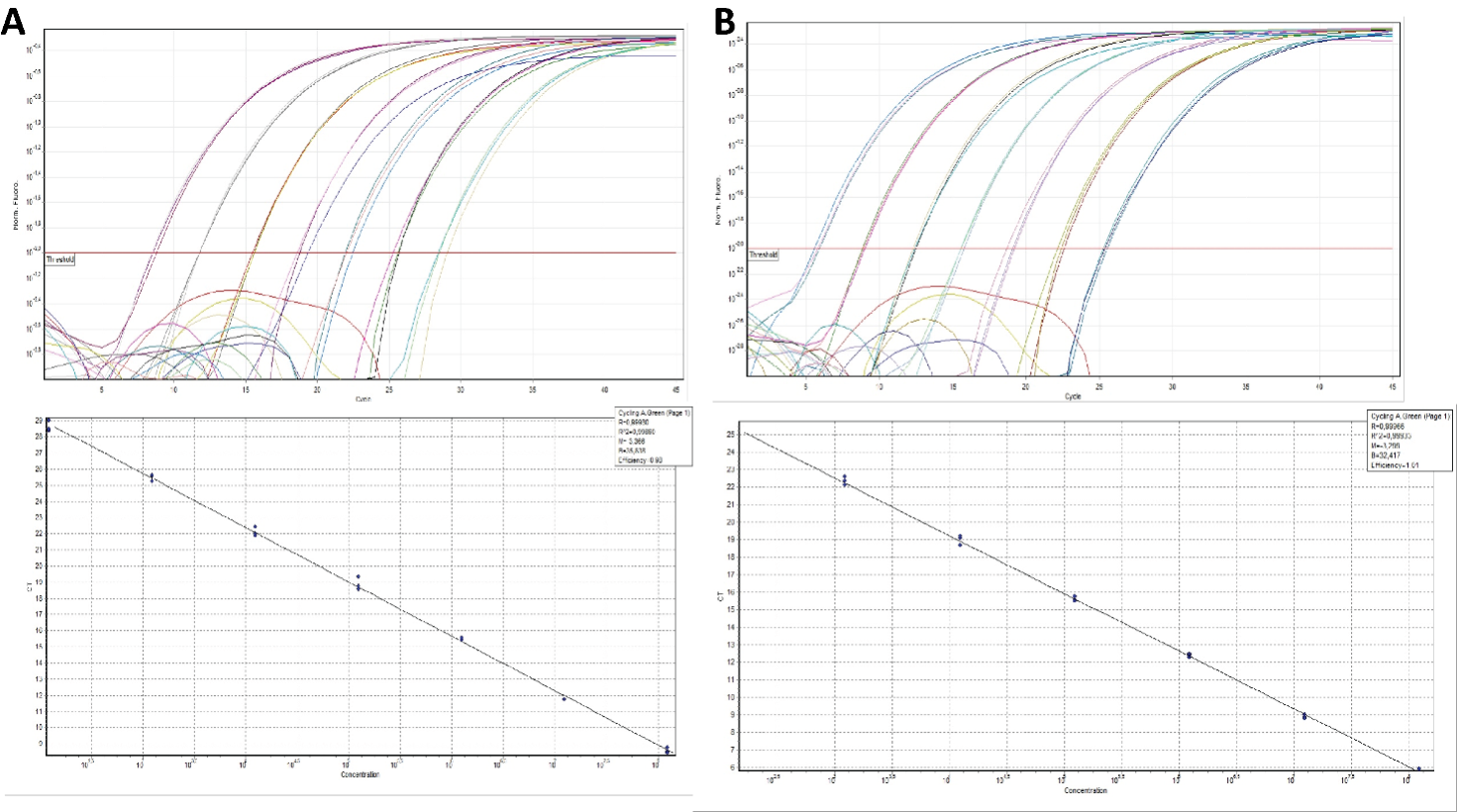


Suppl. Fig. 1. PCR standard curves (top) and reaction efficiency (bottom) of A) *w*Ov*ftsZ* circular plasmid and B) *w*Ov*ftsZ* linearized plasmids diluted 1:10 from 10^7 to 10^1 copies/µL.


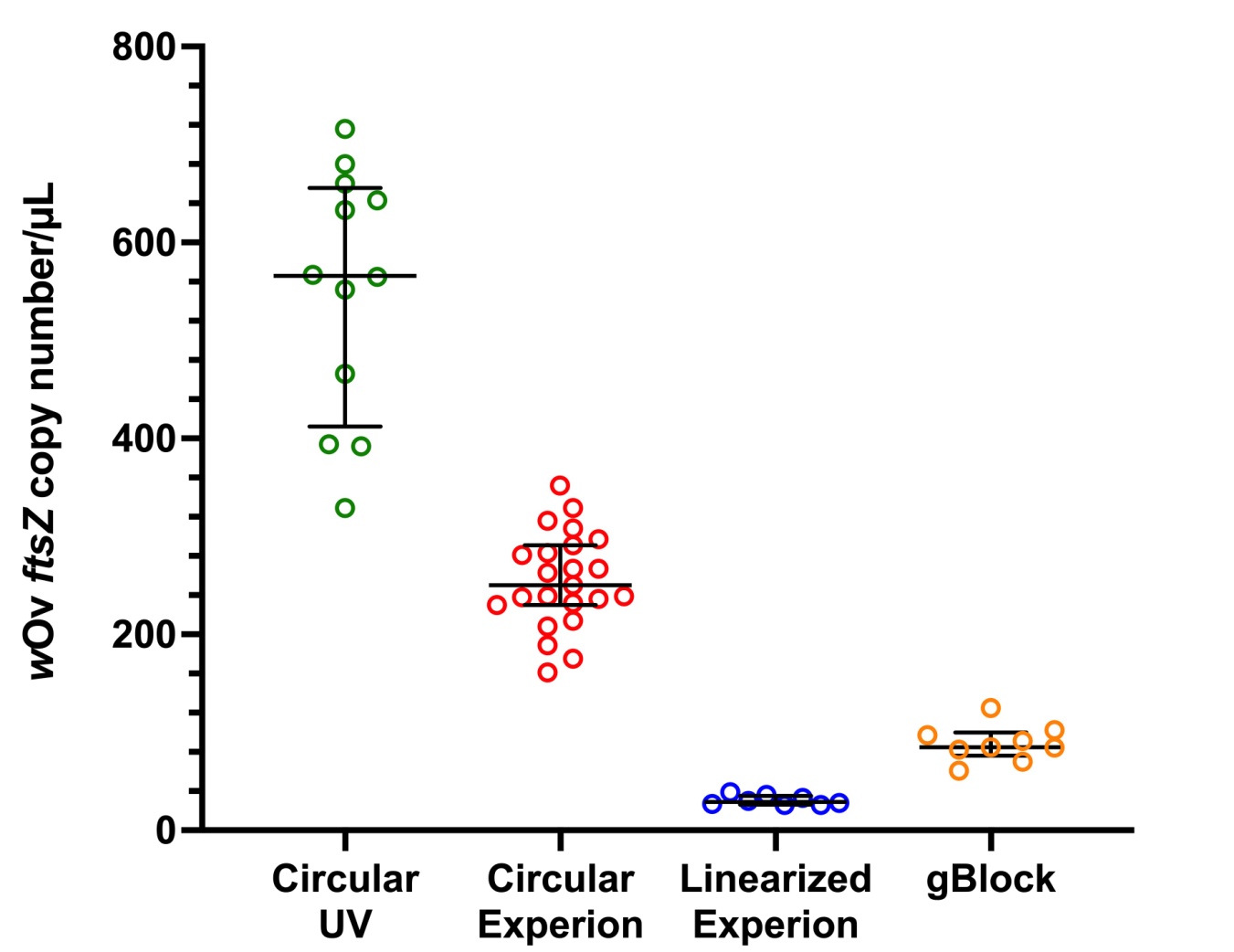


Suppl. Fig. 2Linearized plasmid standard is required for accurate quantification. The plasmid preparation was quantified by two methods (UV spectroscopy and Experion microfluidic assay (BioRad, Feldkirchen, Germany). The *w*Ov*ftsZ* copy numbers from the MF positive control varied by a factor of 2 depending on the DNA quantification method used with higher copy numbers using UV spectroscopy compared to Experion microfluidic assay. Moreover, differences in copy number calculation by usage of supercoiled and linearized plasmid standards were assessed. The plasmid standard was linearized with NotI and linearization was confirmed by gel analysis. The circular plasmid standard and linearized plasmid standard differed by 8-fold. Thus, the supercoiled plasmid standard resulted in an overestimation of *w*Ov*ftsZ* copy numbers. gBlock standards resulted in copy number estimations in same order of magnitude as the linearized plasmid standard.


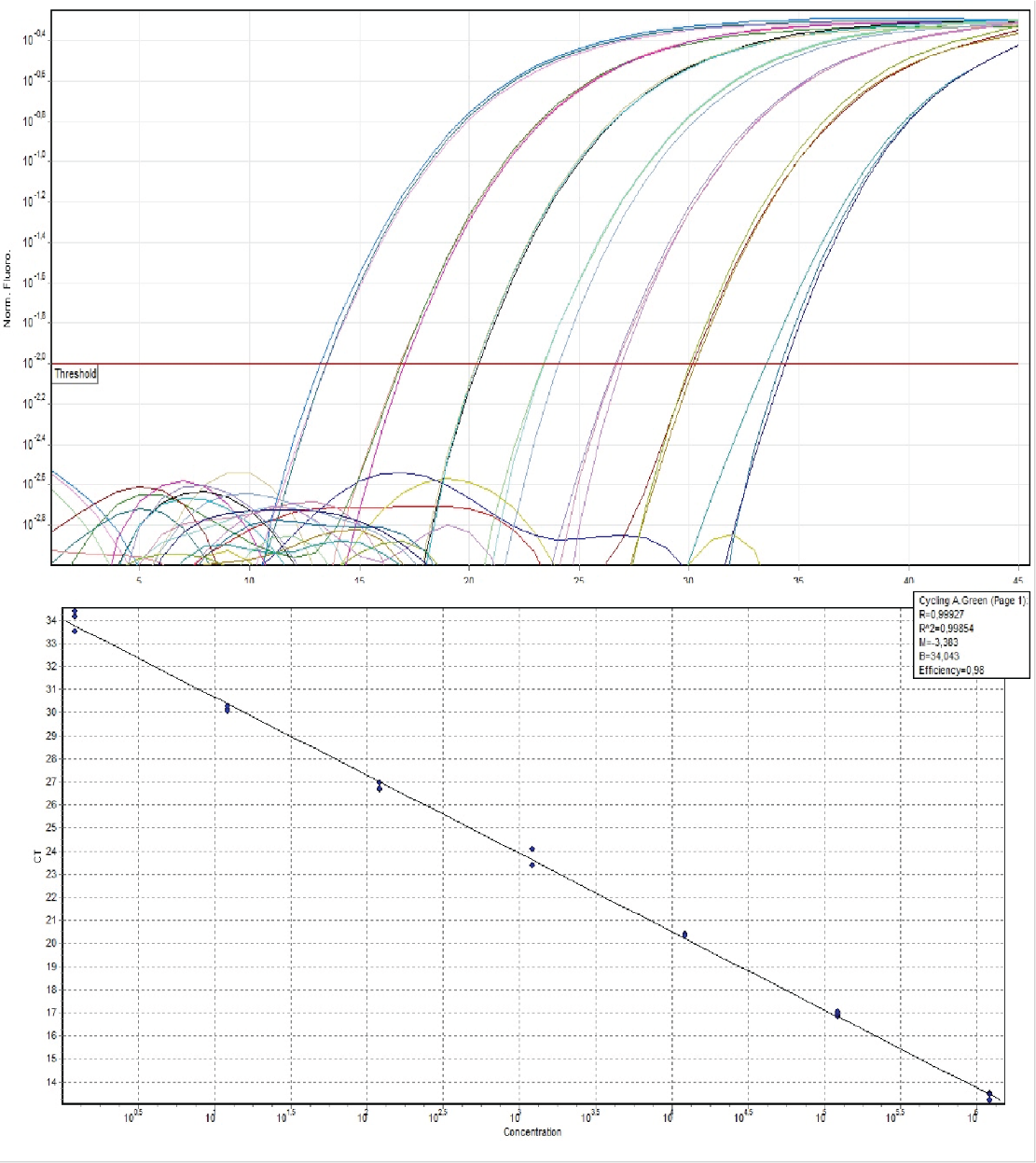


Suppl. Fig. 3. PCR standard curve (top) and reaction efficiency (bottom) using *w*Ov*ftsZ* gBlock® diluted 1:10 from 10^6 to 10^0 copies/µL.

Suppl Table 1 Inter-assay reproducibility of a test series of 10 samples containing approximately 50 MF from *L. sigmodontis*

| **MF/well** | ***w*Ls*ftsZ* Ct** | **SD** | ***ftsZ*/MF** | **LsActin**  **Ct** | **SD** | **LsActin/MF** |
| --- | --- | --- | --- | --- | --- | --- |
| 46 | 26.79 | 0.36 | 8.15 | 23.76 | 0.09 | 483 |
| 61 | 26.8 | 0.1 | 6.15 | 23.49 | 0.36 | 434 |
| 52 | 27.39 | 0.27 | 4.81 | 23.67 | 0.39 | 453 |
| 50 | 27.33 | 0.06 | 5.00 | 23.9 | 0.29 | 404 |
| 51 | 26.91 | 0.07 | 6.86 | 23.48 | 0.15 | 523 |
| 49 | 27.68 | 0.49 | 4.08 | 23.91 | 0.17 | 409 |
| 50 | 28.04 | 0.54 | 3.00 | 23.67 | 0.31 | 470 |
| 51 | 26.49 | 0.33 | 8.82 | 23.66 | 0.15 | 465 |
| 52 | 27.33 | 0.34 | 4.81 | 23.85 | 0.19 | 403 |
| 64 | 26.53 | 0.38 | 7.03 | 23.28 | 0.14 | 478 |

Suppl. Table 2 Inter-assay reproducibility of gBlock based standard curve for *w*Ov*ftsZ*

| **Given copies/µL** | ***w*Ov*ftsZ* Ct**  **run 1** | **SD** | ***w*Ov*ftsZ* Ct**  **run 2** | **SD** | ***w*Ov*ftsZ* Ct**  **run 3** | **SD** | ***w*Ov*ftsZ* Ct**  **run 4** | **SD** | ***w*Ov*ftsZ* Ct**  **run 5** | **SD** |
| --- | --- | --- | --- | --- | --- | --- | --- | --- | --- | --- |
| 1.2*10^5^ | 16.26 | 0.17 | 16.93 | 0.1 | 17.06 | 0.06 | 17.27 | 0.08 | 17.18 | 0.05 |
| 1.2*10^4^ | 19.6 | 0.11 | 20.34 | 0.05 | 20.32 | 0.09 | 20.18 | 0.15 | 20.95 | 0.92 |
| 1.2*10^3^ | 23.14 | 0.07 | 23.61 | 0.39 | 23.8 | 0.03 | 24.1 | 0.22 | 24 | 0.17 |
| 1.2*10^2^ | 26.54 | 0.52 | 26.77 | 0.15 | 27.38 | 0.3 | 27.33 | 0.26 | 27.13 | 0.33 |
| 1.2*10^1^ | 29.97 | 0.36 | 30.16 | 0.11 | 31.02 | 0.37 | 30.75 | 0.34 | 30.31 | 0.35 |
| 1.2*10^0^ | 34.07 | 0.44 | 33.97 | 0.38 | Nd* |  | 34.45 | 1.19 | 34.18 | Nd |

*Not determined, single values
